# Effects of Maternal Nightshift Work on Evening Energy Intake, Diet Quality and Meal Timing in the Family: An Observational Study

**DOI:** 10.3390/nursrep11040077

**Published:** 2021-10-22

**Authors:** Alan Flanagan, Elizabeth Lowson, Bruce A. Griffin, Debra J. Skene

**Affiliations:** 1 Department of Biochemical Sciences, Faculty of Health and Medical Sciences, University of Surrey, Guildford GU2 7XH, UK; d.skene@surrey.ac.uk; 2 Department of Nutritional Sciences, Faculty of Health and Medical Sciences, University of Surrey, Guildford GU2 7XH, UK; b.griffin@surrey.ac.uk; 3 Department of Sociology, Faculty of Arts and Social Sciences, University of Surrey, Guildford GU2 7XH, UK; elizabeth.lowson@hotmail.com

**Keywords:** nightshift, family, meal timing, diet quality, children

## Abstract

The percentage of women working regular nightshift work has increased in the past decade. While nightshift work has the potential to exert adverse effects on dietary habits, little is known about the impact of a parent working nightshifts on dietary habits in the family. We analysed energy intake, meal timing, and diet quality among dependent children and male partners of 20 female UK National Health Service (NHS) nurses working rotational nightshifts. Comparing nightshift against non-nightshift conditions, we hypothesised that maternal nightshift work would affect the evening energy intake, diet quality and time of eating of dependent children and adult partners. Primary outcomes were absolute energy intake and the proportion of daily energy intake consumed in the evening (16:00–23:59 h). Our results show that in pre-teen children aged 8–12 years (*n* = 13, mean ± SD, 9.9 ± 1.6 yrs; 9 males), the proportion of total daily energy intake consumed during periods of nightshift work was significantly greater compared to periods of non-nightshifts (45.7% ± 8.8% vs. 39.7% ± 7.0%, mean ± SD, *p* = 0.012). There was no effect of nightshift work on dietary habits in teenage children or partners. The finding of a greater proportion of daily energy consumed in the evening period in pre-teen children is noteworthy, as it suggests that pre-teen children more dependent than older teenage children may be more vulnerable to disruptions to dietary patterns associated with maternal nightshift work.

## 1. Introduction

A substantial body of research demonstrates the adverse effects of nightshift work on metabolic health [1,2]. Over the past decade, the percentage of women working nightshifts has increased by 12%, with up to 9.7% of women in employment report working regular nightshifts [3]. However, little is known about the impact of nightshift work within a family unit of a parent working nightshifts on meal timing and dietary intake in dependent children and partners. Women of young children remain likely to have a primary role in food preparation within a household [4]. While there is evidence that more strict divisions of gendered household roles have declined over recent decades, in relation to diet and nutrition women remain more likely to have primary responsibility for household meal planning, food preparation, and food shopping [5]. The potential implications of this pattern of employment for within-home dietary intake warrant investigation, particularly with regard to previous research demonstrating the impact of shift work on participation in social and family life, for example missed social gatherings, recreational activities, and children’s events [6].

While the majority of the associations between altered meal patterns, meal timing, and metabolic health risk relate to adults, there is a limited literature in paediatric populations, which suggests that sleep timing and meal timing may be associated with childhood obesity [7,8,9]. An analysis of 12–18-year-old US teenagers stratified by body mass index [BMI] into ‘normal, ‘overweight’ or ‘obese’ categories found that, despite similar patterns of energy intake, children defined as ‘obese’ [BMI > 30 m/kg^2^] consumed a greater proportion of daily energy between 18:00–21:00 h [7]. In pre-teen 6–11-year olds, energy intake in children with obesity was higher between 20:00–22:00 h compared to other categories [7].

Within-home factors, including the activity patterns of parents, may also be relevant in influencing dietary intake in dependent children. Parental activity has previously been identified as a possible within-home factor influencing child sleep timing [9]. There is also evidence to suggest that within-home parental activity factors may influence food intake [8]. The definition of parental activity could also extend to occupational patterns that may require a parent to be away from the home in evening hours, a situation with potential impact on the household responsibilities and duties of dependent children [6]. While these factors may disrupt meal timing and/or dietary composition in dependent children, to the best of our knowledge no study has investigated the effects of maternal nightshift work on dietary intake and timing in dependent children and partners.

The study examined the impact of rotating nightshift work on women NHS nurses and their families, using a mixed methods approach. The study utilised in-depth interviews on the effects of nightshift work within the family, and included endpoint measures of mood ratings, the amount and quality of sleep, and dietary intake. Dietary intake was recorded by each participant, including children, in a 14-day diet diary. The findings in relation to cortisol, mood ratings, and sleep quality, and in relation to energy intake during nightshifts in the nurses, have been previously published [10,11]. Findings in relation to the qualitative data have also previously been published [12]. The present study investigated the impact of nightshift work on evening energy intake, meal timing, and overall diet quality, in dependent children and partners of nurses’ working rotational nightshifts. We hypothesised that children and partners would have different energy intakes, diet quality and meal timing during periods of maternal nightshift work.

## 2. Methods

### 2.1. Participants

Female nurses were recruited through recruiting material distributed in eight NHS Hospital Trusts in Southern England. The study obtained ethical approval from the research governance of each NHS Hospital Trust, the Surrey NHS Research Ethics Committee [REC Ref 06/Q1909/17], and the University of Surrey Ethics Committee [EC/2006/60/SOCIO].

To be eligible for the study, female hospital nurses and midwives (hereafter defined as “nurses”) were required to be aged between 30–55 years, engaged in rotating shifts of no less than 29 h per week, and to be mothers with at least one child aged 8–18 years. Shiftwork schedules of the nurses included a minimum of two consecutive night shifts of at least 8 h between 20:00 and 08:00 h, in addition to day shifts. A total of 11 nurses’ worked three nightshifts, 6 worked two nightshifts, and 2 each worked four and five nightshifts (see Supplementary Table S1 for tabulated nightshift schedule), within the 14-day study period. Exclusion criteria included lack of English fluency and use of sleep medications. Here we present a secondary analysis of data collected during previous research conducted between October 2006–September 2008 at the University of Surrey, into the impact of nightshift work on nurses and their families [12].

### 2.2. Study Design

Participants were provided with a 14-day booklet with individual 24 h diet diaries for each day, and requested to record all food and/or drink intake, the time and location of intake, whether the foods/drinks contained caffeine/alcohol [older children only], who prepared the food, and whether other people were present during the meal. Participants were requested to complete the food diary on the evening of that day’s intake.

### 2.3. Data Analysis

The primary outcome was energy intake in the evening comparing nightshift to non-nightshift conditions. Secondary outcomes included the timing of dinner, time of last recorded calorie intake, and indices of diet quality based on the Alternate Healthy Eating Index 2010 [AHEI-2010]. Children were grouped by age, and divided into ‘pre-teen’ [aged 8–12 years; *n* = 15] and ‘teen’ [aged 12–18 years; *n* = 19] groups.

Nutritics (v5.029, Dublin, Ireland) was used to analyse dietary intake based on data from the 2015 ‘Composition of Foods Integrated Dataset ‘CoFID’ and McCance and Widdowson’s 7th Edition. Demographic averages in grams or millilitres were applied to food diary entries which failed to specify a portion size (i.e., in spoon measures, cups, or grams, or in readily identifiable pre-packaged portions), based on data on the average portion of a given food consumed by males and females across different age ranges from the UK National Diet and Nutrition Survey [13,14]. This approach provided values that were more representative of real intakes compared to UK Food Standards Agency recommended portion sizes. Participants with implausible energy intakes of <400 kcal/day or >4000 kcal/day were excluded from the analysis. This resulted in two out of 15 pre-teen children and one adult male partner out of 20 being excluded from the final analysis. Energy intake was divided into four time-bins, accounting for average school start and finish times in the UK [~08:00–09:00–15:00–16:00 h]; “morning” (06:00–11:00 h); “day” (11:01–15:59 h); “evening” (16:00–23:59 h) and “night” (00:00–05:59 h). Data for the adult male partners were also organised according to this time-bin categorisation. Energy intake was separated into two conditions, ‘nightshift’ and ‘non-nightshift’, and mean energy intake calculated for each time-bin in both conditions. The energy consumed during either condition was expressed as a percentage of total daily energy intake calculated against the total daily energy intake for that calendar day. The percentage value was used to assess changes in the proportion of daily energy consumed by children during the nightshift condition compared to the non-nightshift condition. Absolute energy intake in the evening was calculated as calories [kcal] between 16:00–23:59 h. Last recorded energy intake was defined as the clock time [24 h clock] at which the last calorie intake was recorded in a diet diary, averaged from the three latest recordings for both nightshift and non-nightshift conditions.

Data were analysed using GraphPad Prism v9.1 (GraphPad Software 2021, La Jolla, CA, USA). Data were assessed for normality using the D’Agostino-Pearson test for normality, and checked for outliers by visual inspection of boxplots and ROUT method using a false discovery rate of 1%. There were no outliers removed from the data set. The results of the analyses are presented as means and standard deviations (SD), and 95% confidence intervals [CI] reported with *p*-values of <0.05 on two-tailed tests as the threshold for statistical significance. Cohen’s *d* measure of effect size is also reported. Paired *t*-tests were used to test for differences between nightshift and non-nightshift conditions for all analyses.

## 3. Results

The analysis consisted of 32 dependent children in total, including 19 teenage children (4 pairs of siblings) aged 12–18 years (mean ± SD, 15.5 ± 1.5 yrs; 10 males), and 13 pre-teen children (4 pairs of siblings also) aged 8–12 years (mean ± SD, 9.9 ± 1.6 yrs; 9 males), who completed 14-day dietary records and were included in the analysis. Food diaries from 19 adult male partners (mean ± SD, 44.6 ± 7.2 yrs) were also analysed. A summary of results is presented in Table 1.

### 3.1. Energy Intake

**Figure 1:**
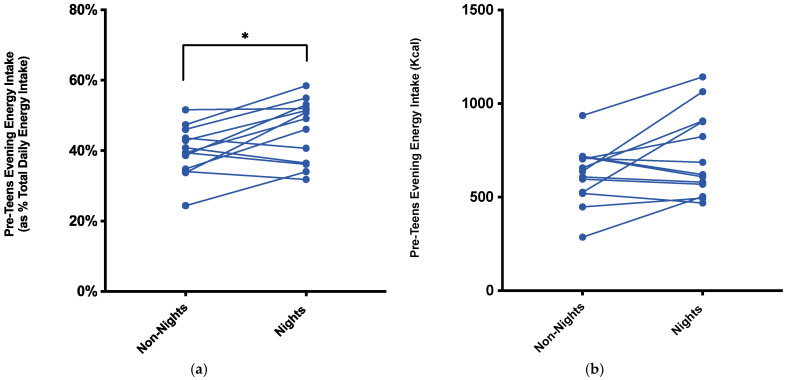
(**a**) The proportion (as a percentage) of total daily energy and (**b**) absolute energy consumed (kcal) by pre-teen children (*n* = 13) in the evening period [16:00–23:59 h] when mothers were working nightshifts vs. not on nightshifts. * *p* = 0.012 for the proportion of total daily energy consumed during the nightshift condition compared to non-nightshifts (paired *t*-test).

In the pre-teen group, total daily energy intake did not differ between the nightshift and non-nightshift conditions (Table 1). Energy intake in the evening [16:00–23:59 h] was higher during nightshifts (720 ± 224 kcal) compared to non-nightshifts (619 ± 157 kcal), an average difference of 100 kcal (95% CI, −7.4–209.2 kcal) which was not statistically significant (*p* = 0.065). The proportion of total daily energy consumed in the evening was higher during nightshifts (45.7% ± 8.8%) compared to non-nightshifts (39.7% ± 7.0%), a statistically significant increase of 6% (95% CI, 1.5–10.5%), *t*(12) = 2.919, *p* = 0.012), *d* = 0.81 (Figure 1).

In the teenage group, there was no significant difference in total daily energy intake, energy consumed in the evening [16:00–23:59 h], or the proportion of daily energy consumed in the evening, comparing the nightshift and non-nightshift conditions (Table 1). In the partners group, there were also no significant differences in total daily energy intake, energy consumed in the evening [16:00–23:59 h], or the proportion of daily energy consumed in the evening, comparing the nightshift and non-nightshift conditions (Table 1).

### 3.2. Meal Timing

**Figure 2:**
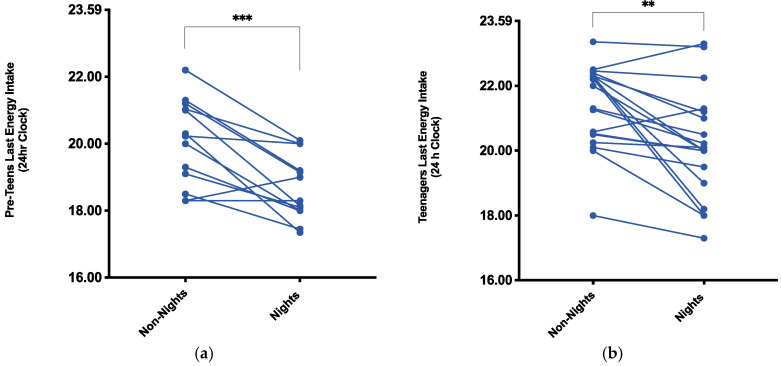
(**a**) Last recorded time of energy intake in the pre-teen group (*n* = 13) and (**b**) the teen group children (*n* = 19) in the evening period [16:00–23:59 h] when mothers were working nightshifts vs. not on nightshifts. *** *p* = 0.0007 and ** *p* = 0.001 for the difference in last recorded energy intake time during the nightshift condition compared to non-nightshifts in pre-teens and teens, respectively (paired *t*-test).

Average dinner time in the pre-teen group was similar between nightshifts and non-nightshifts, approximately 18:15 h (Table 1). However, the pre-teen group consumed their last recorded energy intake during the nightshift condition significantly earlier (18:40 h ± 00:57 h) compared to during the non-nightshift condition (20:03 h ± 01:16 h), a statistically significant difference of 01:22 h (95% CI, 00:42–02:00 h), *t*(12) = 4.542, *p* = 0.0007, *d* = 1.25 (Figure 2).

The teenage group also exhibited a similar average dinner time (~19:43 h) between nightshift and non-nightshift conditions (Table 1). The teenage group, however, also consumed their last recorded energy intake during the nightshift condition earlier (20:09 ± 01:39 h) compared to during the non-nightshift condition (21:24 ± 01:17 h), a statistically significant difference of 01:13 h (95% CI, 00:32–01:55 h), *t*(18) = 3.776, *p* = 0.001, *d* = 0.86 (Figure 2). By contrast, in the partners group, both average dinner time (~18:53 h) and the last recorded energy intake (~19:50 h) were similar between the nightshift and non-nightshift conditions (Table 1).

### 3.3. Diet Quality

There was no significant difference in the modified AHEI-2010 scores for the pre-teen children, comparing nightshifts to non-nightshift conditions (Table 1). In the teenage group, average modified AHEI-2010 scores were higher (indicating better diet quality) during nightshifts (35.1 ± 6.7) compared to non-nightshifts (31.8 ± 3.8), a difference of 3.2 (95% CI, 0.2–6.1) that was statistically significant between conditions *t*(18) = 2.312, *p* = 0.032, *d* = 0.52. Likewise in the partners group, the average modified AHEI-2010 scores were higher (indicating better diet quality) during nightshifts (39.7 ± 6.2) compared to non-nightshifts (38.2 ± 5.9), a difference of 1.5 (95% CI, 0.1–2.8) that was statistically significant between conditions, *t*(18) = 2.379, *p* = 0.029, *d* = 0.55.

## 4. Discussion

The pre-teen and teenage children in this study had mothers who worked rotational shift patterns. The hypothesis was that these children would have changes to their energy intake and diet quality during the periods of their mothers’ night-shift work. In the pre-teen group, there was no significant difference in total energy consumed in the evening, the timing of the main dinner meal, or diet quality scores, between the nightshift and non-nightshift conditions. However, in this pre-teen group the proportion of total daily energy intake (TDEI) consumed in the evening period (16:00–23:59 h) was 6% (95% CI, 1.5–10.5%) higher during the mothers’ nightshifts (45.7% ± 8.8%) compared to non-nightshifts (39.7% ± 7.0%). Furthermore, although not statistically significant, the energy intake was on average 100 kcal higher during the periods of nightshifts. The increase in the proportion of TDEI consumed in the evening, without a concomitant increase in absolute energy intake in the evening, suggests an altered distribution of energy intake during periods of nightshifts. It is also possible that this increase in the proportion of evening energy reflects lower energy intake earlier in the day, and potentially other factors influencing the overall pattern of energy intake outside the evening window may be relevant. For example, it could be that during periods of nightshift work with the mother either being out of the home during the morning or having extended sleep during the day may influence food intake in younger dependent children. Indeed, this period of delegated tasks to partners or older children was a factor disrupting household routines in the original research on these families [12].

For both the pre-teen and teenage groups, the timing of the last recorded energy intake was significantly earlier during the nightshift compared to the non-nightshift condition. The pre-teen group recorded their last energy intake 01:22 h (95% CI, 00:42–02:00 h) earlier during nightshifts compared to non-nightshifts (18:40 h and 20:03 h, respectively). This finding could reflect the fact that nightshift work for the nurses started on average at 20:00 or 21:00 h. The average last recorded energy intake in the pre-teen group occurring at 18:40 h could reflect the mothers’ meal preparations before departing for nightshifts, which was suggested by the original research [12]. For the teenage children, the tendency for later last recorded energy intake compared to the pre-teen children may relate to their greater household independence. Nevertheless, the direction of effect in relation to the teenage children also indicates a 01:13 h (95% CI, 00:32–01:55 h) earlier timing of last recorded energy intake during periods of nightshift work. Whether this also reflects meal preparation for the nightshift period is a matter of speculation.

For the teenage group and the partners group, there were no significant differences in the total evening energy intake, the proportion of energy consumed in the evening, nor in the timing of the main dinner meal between the nightshift and non-nightshift conditions. However, both of these groups showed a statistically significant increase of in AHEI-2010 scores between the nightshift and non-nightshift conditions (31.8 to 35.1 and 38.2 to 39.7 for the teenage and partner groups, respectively). These results, however, should be interpreted with caution in inferring any genuine effect of nightshifts, since while statistically significant a mean difference of 3.3 and 1.5 in the teenage and partner groups, respectively, would not be meaningful differences in a real-life context. Data from the United States has previously shown that average scores of the predecessor Healthy Eating Index for U.S. children was 47–50 out of 100 points, while in the UK Whitehall II cohort average adult AHEI-2010 scores were 48.7 [15,16]. The mean scores of 31.8–35.1 and 38.2–39.7 for the teenage and partner groups, respectively, indicate overall poor diet quality. A number of factors should also be taken into account in interpreting the AHEI-2010 scores. Firstly, industrial trans fats are now largely absent in the UK food supply [17], and as trans fats are scored in the AHEI-2010 as a percentage of total energy under the moderation principle, a maximum score was attained for all participants. In addition, lower sodium intake in the food supply [18] typically corresponded to increased points. Finally, dairy as a food group was added to the original scoring table and, in particular milk and yogurt, contributed substantially to daily scoring which may otherwise have been lower. Thus, the AHEI-2010 scoring amongst the participants in this study largely reflected a default score from diet components under the moderation principle. Under the adequacy principle, only dairy made an impactful contribution to diet quality, and the participants were uniformly characterised by low vegetable intake, low polyunsaturated fats, low long-chain marine omega-3 fatty acids eicosapentaenoic acid (EPA) and docosahexaenoic acid (DHA), low whole fruit, low wholegrains intakes. These dietary characteristics may reflect the pronounced socio-economic barriers in the UK to families accessing healthy dietary patterns. For example, in the UK, households in the lower 50% of income would have to spend 30% of disposable income to meet the Eatwell Guide recommendations, compared to just 12% spend for households in the top 50% of income [19].

Previous research in UK children found that the timing of the evening meal had no impact on mean TDEI [20]. In this analysis, the ‘evening meal’ was designated as the period of highest total energy intake for a child [20]. However, in the UK, a noted feature of population trends in energy consumption is an increase in energy from snacking episodes, and a decline in energy coming from meals [21]. Our data suggest that snacking after the main evening dinner meal was common, and associated with a 01:22 h and 01:13 h later last recorded energy intake in pre-teen and teenage children, respectively. This may be relevant to factors such as family meals, as suggested by a recent systematic review of the literature examining family meals frequency and dietary or weight outcomes, which concluded that there were overall positive associations between family meal frequency and diet quality amongst children and adolescents [22]. However, the review identified that, irrespective of participation in family meals, most adults and children still failed to meet the recommended intakes of healthy foods, with barriers to eating meals together, which could include child age, busy families, or contextual factors in low-socioeconomic households [22]. Given the dietary quality scores in the present study, it is possible that shift work represents another such barrier, being a relatively constant disruption to household food planning and eating patterns.

The strength of the present study is the novel research question, as we are unaware of prior research that has examined the impact of nightshift work on dietary factors within the family. Limitations include the dietary recall records being prone to both under and over reporting [23], and some energy intake may have gone undocumented with participants completing 24 h food recall diaries.

## 5. Conclusions

In conclusion, our data do not suggest that nightshift work impacts on the amount of energy consumed in the evening or the timing of the dinner meal, particularly in adult male partners and teenage children. On the basis of the differences in diet quality between nightshifts and non-nightshifts in our study, it is reasonable to conclude that shift work does not strongly influence diet quality in children or partners. The fact that the timing of the last recorded energy intake was approximately an hour earlier in both pre-teen and teenage children during nightshifts may reflect maternal meal preparations prior to periods of nightshifts. The finding of a greater proportion of daily energy consumed in the evening period in pre-teen children compared to older teenage children is noteworthy, as it suggests that pre-teen children may be more vulnerable to disruptions to dietary patterns associated with maternal nightshift work.

## Figures and Tables

**Table 1:** Dietary Intake of Children During Maternal Nightshift Work vs. Non-Nightshifts.

Pre-teen Children (*n* = 13)	Non-Nightshifts	Nightshifts	p Value
Total Daily Energy, kcal/d	1550 ± 250	1583 ± 385	0.682
Proportion Energy in Evening, %TDEI	39.7% ± 7.0%	45.7% ± 8.8%	0.012 *
Evening Energy, kcals	619 ± 157	720 ± 224	0.065
Dinner Meal Timing, 24 h Clock	18:13 ± 00:46 h	18:19 ± 00:48 h	0.484
Last Energy Intake, 24 h Clock	20:03 ± 01:16 h	18:40 ± 0:57 h	0.0007 ***
AHEI-2010 Score, (Numeric from 110)	27.7 ± 3.3	28.5 ± 3.5	0.416
**Teen Children (*n* = 19)**	**Non-Nightshifts**	**Nightshifts**	***p*** **Value**
Total Daily Energy, kcal/d	1599 ± 498	1582 ± 580	0.906
Proportion Energy in Evening, %TDEI	46.8% ± 9.3%	47.8% ± 13.1%	0.775
Evening Energy, kcals	740 ± 220	756 ± 321	0.826
Dinner Meal Timing, 24 h Clock	19:43 ± 01:01 h	19:43 ± 01:45 h	0.966
Last Energy Intake, 24 h Clock	21:24 ± 01:17 h	20:09 ± 01:39 h	0.001 **
AHEI-2010 Score, (Numeric from 110)	31.8 ± 3.8	35.1 ± 6.7	0.032 *
**Partners (*n* = 19)**	**Non-Nightshifts**	**Nightshifts**	***p*** **Value**
Total Daily Energy, kcal/d	1804 ± 426	1843 ± 532	0.675
Proportion Energy in Evening, %TDEI	48.3% ± 10.5%	48.7% ± 9.6%	0.827
Evening Energy, kcals	848 ± 198	884 ± 303	0.568
Dinner Meal Timing, 24 h Clock	18:51 ± 00:49 h	18:54 ± 00:48 h	0.679
Last Energy Intake, 24 h Clock	19:47 ± 01:16 h	19:53 ± 01:11 h	0.210
AHEI-2010 Score, (Numeric from 110)	38.2 ± 5.9	39.7 ± 6.2	0.028 *

* *p* ≤ 0.05; ** *p* ≤ 0.01; *** *p* ≤ 0.001. Kcal = kilocalories; TDEI = total daily energy intake; % = proportion of total daily energy as a percentage; AHEI-2010 = Alternate Healthy Index 2010.

## Data Availability

The data presented in this study are available on request from the corresponding author.

## Supplementary Materials

The following are available online at https://www.mdpi.com/article/10.3390/nursrep11040077/s1, Table S1: Shift Pattern of Nurses.
