# Supplementary material for: Effects of Maternal Nightshift Work on Evening Energy Intake, Diet Quality and Meal Timing in the Family: An Observational Study
Source: Nurs Rep. 2021 Oct 22;11(4):823–31. doi: 10.3390/nursrep11040077 (PMC8715464; doi:10.3390/nursrep11040077)
Supplement: Supplementary file 1 [file nursrep-11-00077-s001.zip › nursrep-1401188-supplementary.pdf]

Effects of Maternal Nightshift Work on Evening Energy Intake, Diet Quality and Meal Timing in the Family: An Observational Study

Supplementary Data

Table S1: Shift Pattern of Nurses

Famiy	Day 1	Day 2	Day 3	Day 4	Day 5	Day 6	Day 7	Day 8	Day 9	Day 10	Day 11	Day 12	Day 13	Day 14
A	NN	NN	N	N	N	NN	NN	NN	NN	NN	NN	NN	NN	NN
B	NN	NN	NN	NN	A	N	N	N	NN	NN	NN	NN	NN	NN
C	NN	NN	S	NN	NN	N	N	N	NN	NN	NN	ANN	NN	NN
D	NN	N	N	N	NN	NN	NN	NN	NN	N	NN	NN	NN	NN
E	NN	NN	NN	N	N	NN	NN	NN	NN	NN	NN	NN	NN	NN
F	MN	NN	NN	NN	M	NN	NN	NN	N	N	NN	NN	NN	NN
G	NN	NN	M	NN	N	N	NN	NN	NN	NN	S	NN	NN	NN
H	NN	N	N	N	NN	NN	NN	NN	NN	NN	NN	NN	NN	NN
I	NN	NN	NN	NN	NN	N	N	N	NN	NN	NN	S	M	S
J	NN	S	NN	NN	N	N	N	NN	NN	NN	NN	NN	NN	NN
K	NN	NN	NN	N	N	N	NN	NN	NN	NN	NN	NN	NN	NN
L	NN	NN	NN	NN	N	N	N	NN	NN	NN	NN	S	N	N
M	NN	NN	TW	NN	NN	NN	NN	N	N	NN	NN	NN	NN	NN
N	NN	NN	NN	N	N	NN	NN	NN	NN	NN	NN	NN	NN	NN
O	NN	NN	NN	NN	NN	NN	NN	N	N	N	N	N	NN	NN
P	NN	NN	N	N	NN	NN	NN	NN	NN	NN	NN	NN	NN	NN
Q	NN	MN	N	N	N	NN	M	NN	M	NN	NN	NN	NN	M
R	NN	N	N	NN	NN	N	NN	NN	NN	NN	NN	NN	NN	NN
S	ANN	ANN	M	NN	NN	NN	NN	NN	NN	NN	N	N	N	NN
T	NN	NN	N	NN	N	N	NN	N	NN	NN	NN	NN	NN	NN

Note: N=Night shift; NN = Non-night shift.
